# ABCD1-Related Disease Presenting as an Upper Motor Neuron-Predominant Amyotrophic Lateral Sclerosis Mimic in a Colombian Female Heterozygote: A Case Report

**DOI:** 10.7759/cureus.114605

**Published:** 2026-08-16

**Authors:** Cristian Correa-Arrieta, Sandra Milena Castellar Leones, Mariana Bernal-Vergara, Mariana Saiz-Briceño, Jorge Diaz-Ruiz, Fernando Ortiz-Corredor

**Affiliations:** 1 Neurology, Centro de Investigación en Fisiatría y Electrodiagnóstico (CIFEL), Bogota, COL; 2 Neurology, Medicina Integral de Precision, Pereira, COL; 3 Physical Medicine and Rehabilitation, Universidad Nacional de Colombia, Bogota, COL; 4 Physical Medicine and Rehabilitation, Centro de Investigación en Fisiatría y Electrodiagnóstico (CIFEL), Bogota, COL; 5 Physical Medicine and Rehabilitation, Hospital Universitario Nacional de Colombia, Bogota, COL; 6 Faculty of Medicine, Institución Universitaria Visión de las Américas, Pereira, COL; 7 Faculty of Medicine, Universidad de la Sabana, Chia, COL; 8 Rehabilitation Medicine, Universidad Nacional de Colombia, Bogota, COL

**Keywords:** abcd1-related disease, adrenomyeloneuropathy, amyotrophic lateral sclerosis mimic, female heterozygote, upper motor neuron syndrome

## Abstract

We report the case of a 47-year-old Colombian woman with a persistent gait disorder beginning at 46 years of age. Neurological examination showed preserved strength, lower-limb spasticity, diffuse hyperreflexia, brisk jaw jerk, and a unilateral Hoffmann sign, without definite bulbar, respiratory, cognitive, sensory, or cerebellar involvement. Brain MRI showed nonspecific occipital/subcortical T2/FLAIR (fluid-attenuated inversion recovery) white matter hyperintensities, and spinal MRI showed mild degenerative changes without compressive myelopathy or spinal cord signal abnormality. Laboratory evaluation did not identify an inflammatory, infectious, nutritional, metabolic, or endocrine cause of myelopathy. Two electrodiagnostic studies showed preserved motor and sensory nerve conduction, normal late responses, normal motor unit action potentials on needle electromyography, and no evidence of active denervation. Neuromuscular ultrasound was also normal, without nerve enlargement, abnormal muscle echogenicity, atrophy, or fasciculations. Overall, there was no diffuse, progressive, multiregional lower motor neuron pattern, and Gold Coast criteria for amyotrophic lateral sclerosis were not fulfilled. A motor neuron disease gene panel identified a heterozygous pathogenic ABCD1 variant, c.1415_1416delAG, p.Gln472Argfs*83. Repeat expansion testing for C9ORF72, ATXN1, and ATXN2 was normal. Classical plasma very-long-chain and branched-chain fatty acid testing showed elevated C26:0 and increased C24:0/C22:0 and C26:0/C22:0 ratios, with normal phytanic and pristanic acids. The clinical, imaging, electrodiagnostic, biochemical, and molecular findings supported ABCD1-related disease with an adrenomyeloneuropathy-predominant phenotype as the most likely explanation for this upper motor neuron-predominant amyotrophic lateral sclerosis (ALS) mimic. This case highlights the importance of considering ABCD1-related disease in adult women with unexplained noncompressive upper motor neuron syndromes when electrodiagnostic studies do not support ALS.

## Introduction

X-linked adrenoleukodystrophy (X-ALD) is an ABCD1-related peroxisomal disorder caused by pathogenic variants in the ABCD1 gene. ABCD1 encodes a peroxisomal membrane transporter involved in the import of activated very-long-chain fatty acids for peroxisomal beta-oxidation. Impaired ABCD1 function results in the accumulation of very-long-chain fatty acid-containing lipids in plasma and tissues. Although the mechanisms linking this biochemical abnormality to neurological injury remain incompletely understood, altered lipid homeostasis, oxidative and mitochondrial stress, neuroinflammation, and progressive axonal degeneration are thought to contribute to the cerebral and spinal manifestations of the disease. The clinical spectrum includes inflammatory cerebral disease, primary adrenal insufficiency, and adrenomyeloneuropathy [1-3]. Though X-ALD has traditionally been recognized as a disorder affecting hemizygous men, heterozygous women may develop an adult-onset, slowly progressive myelopathy characterized predominantly by gait impairment and lower-limb spasticity, sometimes accompanied by sensory symptoms or bladder and bowel dysfunction. In contrast, inflammatory cerebral disease and primary adrenal insufficiency are uncommon in heterozygous women. Diagnosis requires the integration of the clinical phenotype with ABCD1 molecular testing and biochemical biomarkers, including C26:0-lysophosphatidylcholine and/or conventional plasma very-long-chain fatty acid analysis [1-3]. C26:0-lysophosphatidylcholine has superior diagnostic performance because it detects abnormal C26:0-containing lipids more reliably and is less likely than conventional plasma very long-chain fatty acid (VLCFA) analysis to yield false-negative results, particularly in heterozygous women. Therefore, the specific biochemical assay used should be reported whenever available [3].

Heterozygous women should not be regarded merely as asymptomatic carriers. A substantial proportion develop neurological manifestations, including gait impairment, lower-limb spasticity, bladder or bowel dysfunction, sensory symptoms, neuropathic pain, peripheral neuropathy, falls, and reduced quality of life [4,5]. The neurological course is generally chronic and slowly progressive, while spinal MRI may be normal or show only nonspecific abnormalities. Consequently, the differential diagnosis includes structural myelopathy, hereditary spastic paraplegia, degenerative or inflammatory myelopathy, multiple sclerosis, nutritional myelopathies, copper deficiency myelopathy, human T-lymphotropic virus 1 (HTLV-1)-associated myelopathy, HIV-associated myelopathy, primary lateral sclerosis, amyotrophic lateral sclerosis, and other motor neuron disease mimics [4-6].

ABCD1-related myeloneuropathy may mimic an upper motor neuron-predominant form of amyotrophic lateral sclerosis because the affected women can present with progressive gait impairment, lower-limb spasticity, diffuse hyperreflexia, and other corticospinal signs, sometimes without prominent sensory or sphincter symptoms and despite noncompressive spinal imaging. However, the absence of definite lower motor neuron involvement on clinical and serial electrodiagnostic examinations should prompt consideration of alternative diagnoses, including ABCD1-related disease. Distinguishing these conditions early is clinically important because an amyotrophic lateral sclerosis (ALS) diagnosis carries substantially different prognostic and counseling implications. Recognition of ABCD1-related disease redirects the clinical approach toward biochemical and molecular confirmation, symptomatic management, rehabilitation, genetic counseling, cascade testing of at-risk relatives, and phenotype-appropriate longitudinal surveillance. Therefore, ABCD1-related disease should be considered in adult women with chronic noncompressive myelopathy or unexplained upper motor neuron signs, particularly when structural imaging and electrodiagnostic studies do not support a compressive, inflammatory, infectious, nutritional, metabolic, or classical lower motor neuron disorder [2,4-6].

This report describes a symptomatic Colombian woman heterozygous for the pathogenic ABCD1 variant c.1415_1416delAG, p.Gln472Argfs*83, who presented with an upper motor neuron-predominant syndrome resembling an amyotrophic lateral sclerosis-like or primary-lateral-sclerosis-like phenotype. This case highlights the importance of considering ABCD1-related disease in adult women with chronic noncompressive upper motor neuron-predominant syndromes, particularly when serial electrodiagnostic studies do not demonstrate the lower motor neuron involvement required to support ALS.

## Case presentation

A 47-year-old Colombian woman was evaluated in a private neurology outpatient clinic in November 2025 for a persistent gait disorder. In October 2023, she developed pain involving the proximal and distal segments of the left lower limb, which worsened with walking and was partially relieved by rest. She was initially treated with temporary immobilization for suspected knee bursitis and subsequently reported a subjective reduction in muscle bulk and tone, with partial improvement after physical therapy. In January 2024, she experienced recurrent worsening of left lower-limb motor function after bending forward, followed by persistent gait limitation and functional impairment. At the time of neurological evaluation, she required a four-point cane for ambulation but reported no recent falls.

Family history was negative for X-linked adrenoleukodystrophy, progressive spastic gait, unexplained neurological disability, adrenal insufficiency, leukodystrophy, or a known ABCD1-related disorder. Family segregation analysis was not available at the time of manuscript preparation.

Neurological examination showed preserved cognition and cranial nerve function, normal tongue bulk without fasciculations, and preserved muscle strength. Tone was increased in the lower limbs, corresponding to a Modified Ashworth Scale grade of 1. Deep tendon reflexes were 3+/4+ in both the upper and lower limbs, with diffuse hyperreflexia, a brisk jaw jerk, and a left Hoffmann sign. Plantar responses were bilaterally equivocal, without a reproducible extensor response, and were therefore not considered diagnostic of corticospinal tract involvement. Sensory and cerebellar examinations were unremarkable, and there was no definite bulbar or clinically evident respiratory involvement. Overall, the combination of lower-limb spasticity, diffuse hyperreflexia, a brisk jaw jerk, and a unilateral Hoffmann sign supported an upper motor neuron-predominant syndrome without definite clinical evidence of lower motor neuron involvement.

**Figure 1:**
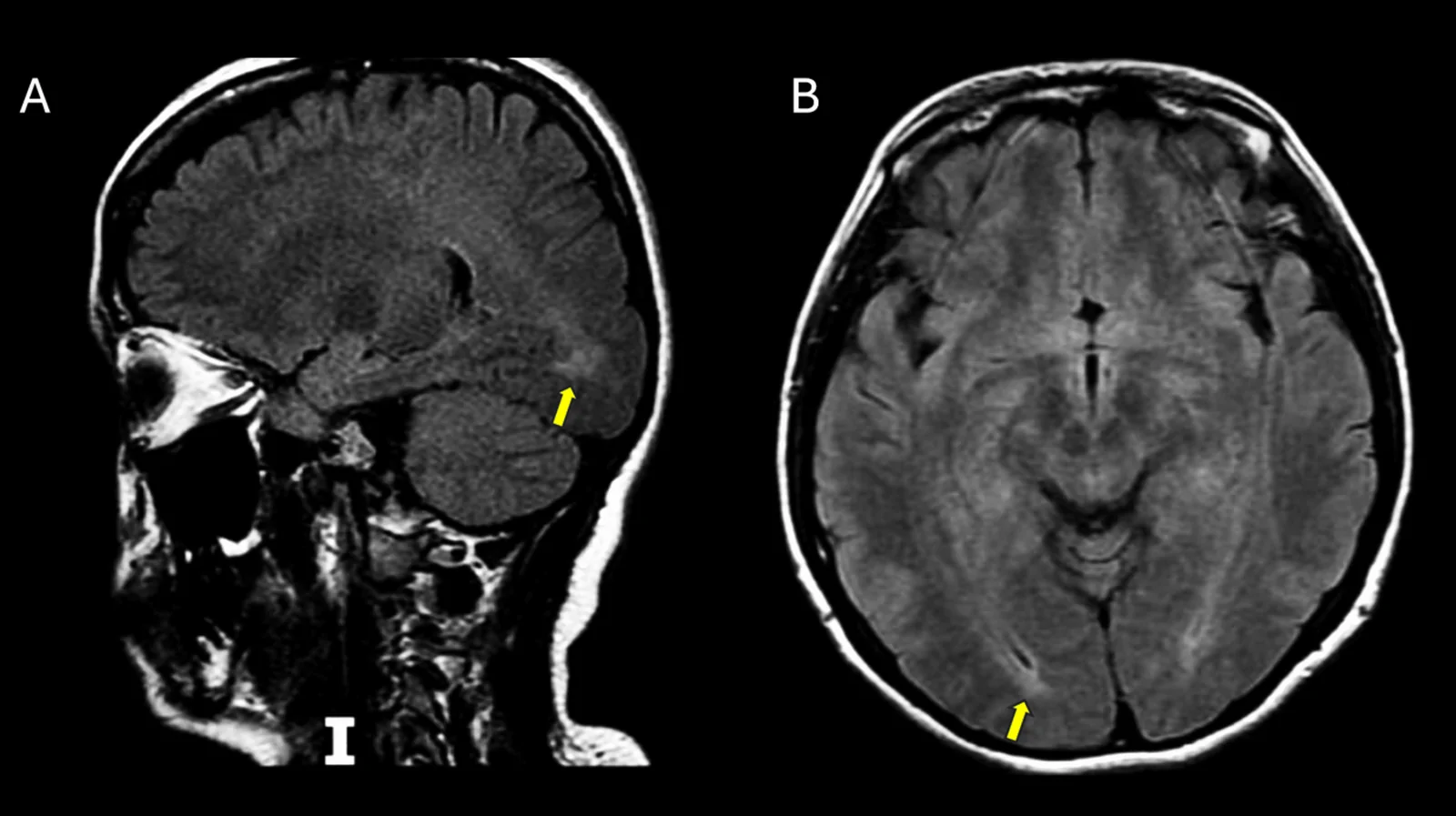
Brain MRI findings. (A) Sagittal FLAIR and (B) axial FLAIR images showing nonspecific occipital/subcortical white matter hyperintensities. Yellow arrows indicate areas of abnormal signal. FLAIR: Fluid-attenuated inversion recovery.

Brain MRI showed nonspecific T2/FLAIR (fluid-attenuated inversion recovery) white matter hyperintensities, most prominent in the occipital and subcortical white matter (Figure 1). Cervical, thoracic, and lumbosacral spine MRI demonstrated mild degenerative changes, including cervical foraminal stenosis, without compressive myelopathy, spinal cord signal abnormality, or clinically significant radiculopathy. Although supratentorial abnormalities may occur within the broader X-ALD (adrenoleukodystrophy) spectrum, the patient’s brain MRI findings were nonspecific and insufficient to establish cerebral X-ALD.

Laboratory evaluation did not identify an inflammatory, infectious, nutritional, metabolic, or endocrine cause of myelopathy. Thyroid-stimulating hormone, serum creatinine, vitamin B12, copper, ceruloplasmin, C3, C4, C-reactive protein, erythrocyte sedimentation rate, dilute Russell viper venom time, prothrombin time, partial thromboplastin time, basal cortisol, 60-minute post-ACTH (adrenocorticotropic hormone) cortisol, and ACTH were within their respective reference ranges. HIV, HTLV-1, syphilis serology, rheumatoid factor, extractable nuclear antigen antibodies, antinuclear antibodies, and anti-double-stranded DNA antibodies were negative. Anti-thyroid peroxidase antibodies were elevated, without biochemical evidence of thyroid dysfunction.

**Figure 2:**
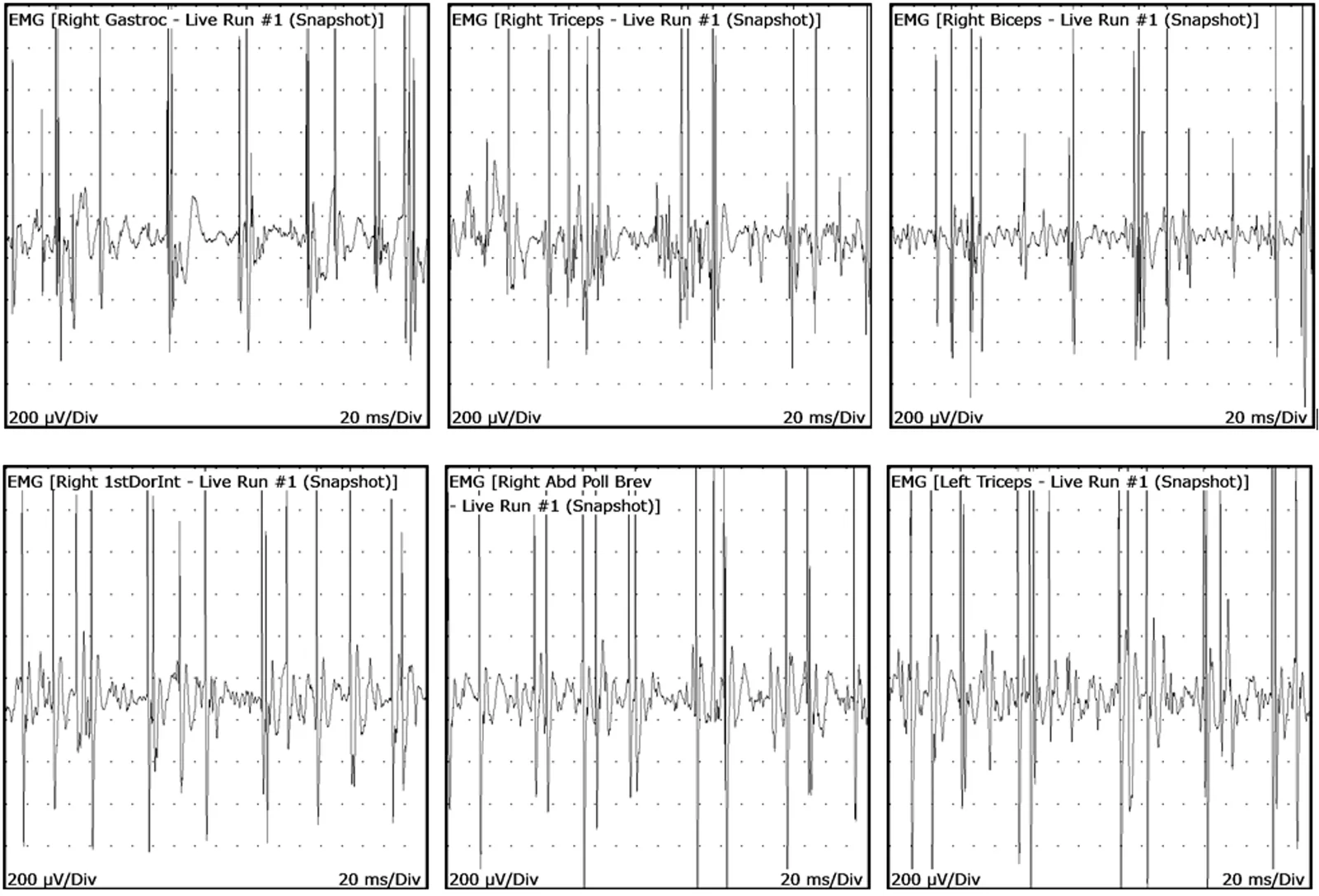
Representative needle electromyography findings. Needle EMG recordings showing normal motor unit action potentials during voluntary activation, without evidence of active denervation.

**Figure 3:**
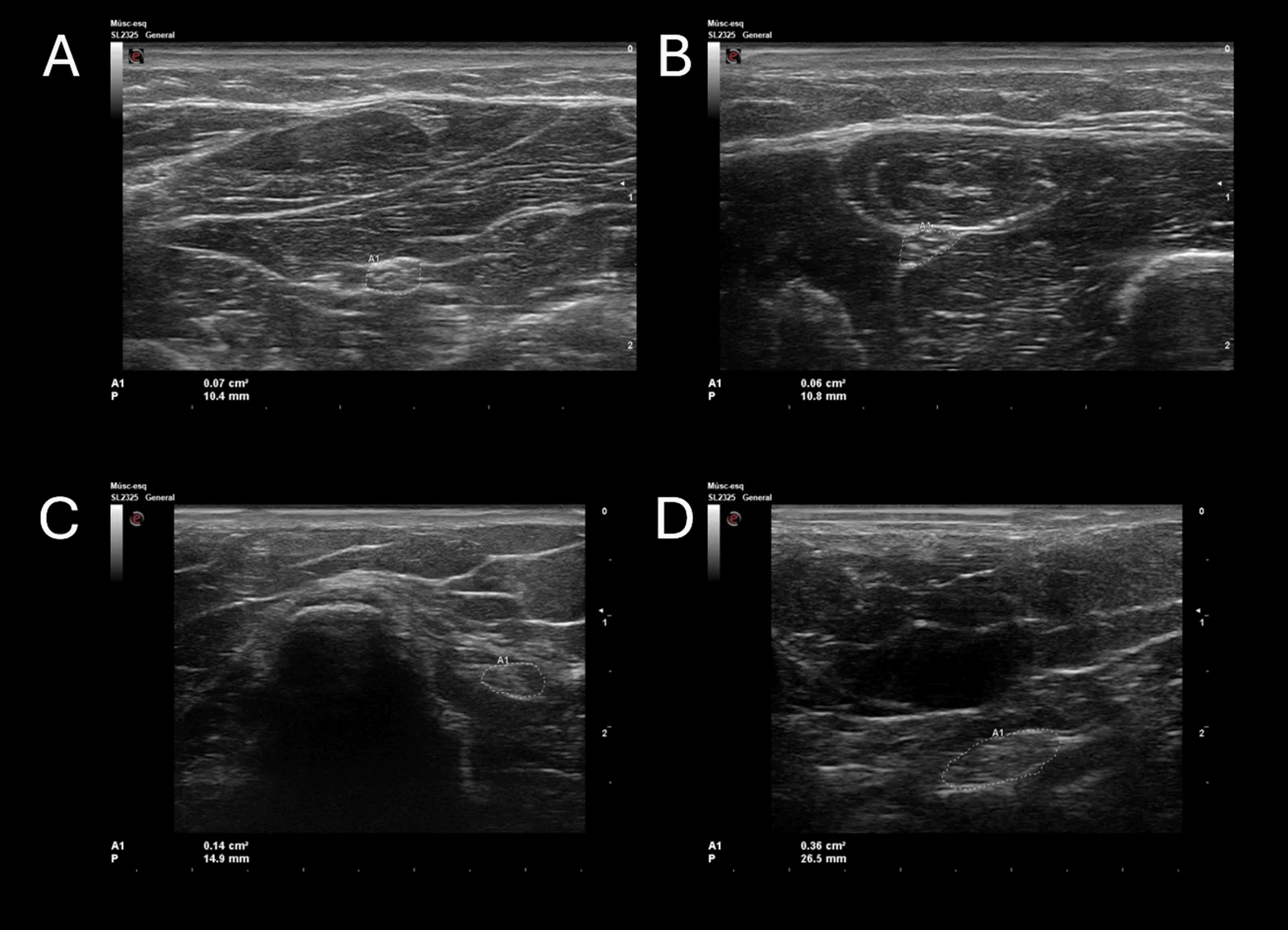
Normal neuromuscular ultrasound of peripheral nerves and adjacent muscles. Neuromuscular ultrasound showing normal nerve morphology and cross-sectional area of the right median nerve in the forearm (A), right ulnar nerve in the forearm (B), right fibular motor nerve at the knee (C), and right tibial nerve at the knee (D). Adjacent muscles showed no sonographic neuropathic or ALS-compatible changes, including no abnormal echogenicity, atrophy, or fasciculations. ALS: Amyotrophic lateral sclerosis.

Two electrodiagnostic evaluations were performed approximately 18 months apart. The first, performed on March 22, 2024, included motor and sensory nerve conduction studies, needle electromyography, and late responses and was interpreted as normal. Submaximal voluntary activation was observed in distal lower-limb muscles in association with increased spastic tone, without evidence of active denervation or neurogenic motor unit abnormalities. The second evaluation, performed on September 11, 2025, included motor and sensory nerve conduction studies, F-wave assessment, needle electromyography, and neuromuscular ultrasound. Motor and sensory nerve conduction studies and F-wave responses were normal, and needle electromyography showed normal motor unit action potentials without fibrillation potentials, positive sharp waves, fasciculations, or other active denervation changes (Figure 2). Neuromuscular ultrasound was also normal, without nerve enlargement, abnormal muscle echogenicity, atrophy, or fasciculations (Figure 3). Detailed quantitative findings from the September 2025 electrodiagnostic and neuromuscular ultrasound evaluation are provided in Supplementary Material 1 (Table 2). Taken together with the absence of definite clinical lower motor neuron signs, the serial electrodiagnostic findings did not demonstrate lower motor neuron involvement or support a diffuse, progressive, multiregional motor neuron disorder; therefore, Gold Coast criteria for ALS were not fulfilled.

Given the chronic noncompressive upper motor neuron-predominant syndrome and the absence of electrodiagnostic findings supporting a classical motor neuron disorder, genetic testing was pursued. A motor neuron disease gene panel identified the heterozygous pathogenic ABCD1 variant c.1415_1416delAG, p.Gln472Argfs*83 (rs387906494). Repeat expansion testing for C9ORF72, ATXN1, and ATXN2 was normal. Conventional plasma very-long-chain and branched-chain fatty acid analysis showed an elevated C26:0 concentration and increased C24:0/C22:0 and C26:0/C22:0 ratios, whereas phytanic and pristanic acid concentrations were within their respective reference ranges (Table 1). C26:0-Lysophosphatidylcholine, which has superior diagnostic performance compared with conventional plasma very long-chain fatty acid (VLCFA) analysis, particularly in heterozygous women, was not documented as having been measured. Consequently, the biochemical evaluation was limited to the less sensitive conventional VLCFA assay. Nevertheless, the concordant abnormalities in C26:0 and both VLCFA ratios provided supportive biochemical evidence when interpreted together with the compatible clinical phenotype and the pathogenic ABCD1 variant.

**Table 1:** Plasma very-long-chain and branched-chain fatty acid testing

Biomarker	Result	Reference range	Units	Interpretation
C22:0, behenic acid	14.86	8.43-33.51	µg/mL	Normal
C24:0, lignoceric acid	21.08	6.87-28.31	µg/mL	Normal
C26:0, hexacosanoic acid	0.56	0.05-0.41	µg/mL	Elevated
C26:1, hexacosenoic acid	0.20	0.05-0.36	µg/mL	Normal
C22:1(n-9), erucic acid	0.37	0.05-2.94	µg/mL	Normal
C24:0/C22:0 ratio	1.42	0.64-1.04	Ratio	Elevated
C26:0/C22:0 ratio	0.038	0.002-0.018	Ratio	Elevated
Phytanic acid	1.26	0.05-3.00	µg/mL	Normal
Pristanic acid	0.11	0.01-0.30	µg/mL	Normal

Taken together, the upper motor neuron-predominant phenotype, absence of compressive myelopathy, serial electrodiagnostic studies that did not demonstrate the lower motor neuron involvement required to fulfill Gold Coast criteria for ALS, pathogenic heterozygous ABCD1 variant, abnormal conventional plasma VLCFA profile, and normal adrenal evaluation supported ABCD1-related disease with an adrenomyeloneuropathy-predominant phenotype as the most likely explanation for the patient’s clinical presentation.

Baclofen was initiated for symptomatic treatment of spasticity but was not tolerated and was subsequently discontinued. During longitudinal follow-up, the patient showed no significant neurological or functional deterioration. Management continued with physical rehabilitation, gait-safety strategies, neurological surveillance, and genetic counseling addressing familial implications and cascade testing.

## Discussion

This case underscores the diagnostic importance of recognizing ABCD1-related disease with an adrenomyeloneuropathy-predominant phenotype as an actionable genetic-metabolic mimic of motor neuron disease in adult women. The patient exhibited a predominantly upper motor neuron phenotype, characterized by gait impairment, lower-limb spasticity, diffuse hyperreflexia, brisk jaw jerk, and a unilateral Hoffmann sign. Serial electrodiagnostic studies did not reveal active denervation, progressive lower motor neuron involvement, or a diffuse multiregional lower motor neuron pattern. In the context of noncompressive spinal imaging, abnormal plasma VLCFA biomarkers, and a pathogenic heterozygous ABCD1 c.1415_1416delAG, p.Gln472Argfs*83 variant, the findings supported ABCD1-related disease with an adrenomyeloneuropathy-predominant phenotype as the most likely explanation for the patient’s upper motor neuron-predominant syndrome rather than classical amyotrophic lateral sclerosis. These findings support the inclusion of ABCD1-related disease in the differential diagnosis of adult women with unexplained chronic noncompressive myelopathy or upper motor neuron-predominant syndromes, particularly when routine structural imaging and serial electrodiagnostic studies are nondiagnostic [2,4-6].

From a neuromuscular perspective, this case delineates a critical diagnostic boundary between ALS, primary-lateral-sclerosis-like phenotypes, and actionable ALS mimics. While gait impairment and upper motor neuron signs may suggest motor neuron disease, these features alone are insufficient to establish ALS in the absence of definitive lower motor neuron involvement. In this patient, preserved strength, absence of bulbar and clinically significant respiratory involvement, lack of active denervation on serial electromyography, and identification of an alternative metabolic-genetic disorder collectively argued against classical ALS. This distinction aligns with current diagnostic frameworks, which require progressive motor impairment with evidence of both upper and lower motor neuron dysfunction after excluding alternative disorders that may better account for the clinical presentation [7].

The Gold Coast criteria for ALS were not fulfilled in this patient. Two electrodiagnostic studies showed preserved motor and sensory nerve conduction, normal F-wave/late responses, normal motor unit action potentials on needle electromyography, and no evidence of fibrillation potentials, positive sharp waves, fasciculations, or other active denervation changes. Neuromuscular ultrasound was also normal, without nerve enlargement or ALS-compatible muscle changes. Therefore, the electrodiagnostic and ultrasound findings did not support lower motor neuron involvement or a diffuse, progressive, multiregional motor neuron disorder [7].

The value of this report is phenotypic and diagnostic rather than molecular. The ABCD1 c.1415_1416delAG, p.Gln472Argfs*83 variant is a recurrent pathogenic frameshift previously documented in X-linked adrenoleukodystrophy and adrenomyeloneuropathy [8-11]. It has been described among the most frequently reported pathogenic ABCD1 variants [11]. A previous report of a symptomatic female heterozygote carrying this variant described a different clinical presentation characterized by prominent cognitive decline, paraspasticity, sphincter dysfunction, and multisystem involvement [8]. In contrast, the current patient developed symptoms in mid-adulthood and exhibited a predominantly spinal upper motor neuron syndrome, preserved cognition, no biochemical evidence of adrenal insufficiency, and no prominent multisystem involvement. This phenotypic variation broadens the recognized clinical spectrum among symptomatic heterozygous women and reinforces that the clinical course of X-ALD cannot be reliably predicted from the specific ABCD1 variant alone [11].

This case has practical diagnostic and clinical implications. In women presenting with chronic spastic paraparesis or an upper motor neuron-predominant syndrome, ABCD1-related disease should be considered after appropriate evaluation for structural, inflammatory, infectious, nutritional, metabolic, and hereditary causes of myelopathy. Relevant differential diagnoses include compressive myelopathy, multiple sclerosis, HTLV-1-associated myelopathy, HIV-associated myelopathy, syphilitic myelopathy, vitamin B12 deficiency, copper deficiency, hereditary spastic paraplegia, primary lateral sclerosis, and other ALS mimics [6]. Hereditary spastic paraplegia remained an important consideration in this patient because chronic spastic gait, preserved strength, predominantly upper motor neuron findings, and noncompressive spinal imaging may occur in both conditions. However, ABCD1-related disease was considered more likely because the pathogenic ABCD1 variant was accompanied by concordant biochemical evidence of impaired VLCFA metabolism, including elevated C26:0 and increased C24:0/C22:0 and C26:0/C22:0 ratios, providing a disease-specific explanation for the clinical phenotype. Nevertheless, because a dedicated hereditary spastic paraplegia gene panel was not performed, selected hereditary spastic paraplegia (HSP)-associated disorders and the possibility of a dual molecular diagnosis cannot be completely excluded. In adult women with unexplained spastic gait, hyperreflexia, noncompressive spinal imaging, and electrodiagnostic findings not supportive of ALS, biochemical testing with plasma very-long-chain fatty acids or C26:0-lysophosphatidylcholine, together with ABCD1 sequencing, should be considered [2,3]. Although C26:0-lysophosphatidylcholine has greater diagnostic sensitivity in women with X-ALD, conventional plasma very-long-chain and branched-chain fatty acid testing was abnormal in this patient and provided supportive biochemical evidence when interpreted in conjunction with the pathogenic ABCD1 variant and compatible clinical phenotype [3]. Biochemical and molecular confirmation redirects the clinical approach from an ALS-centered diagnosis and prognosis toward symptomatic management, rehabilitation, longitudinal surveillance, and family counseling. Management may include treatment of spasticity and pain, physical therapy, gait-safety interventions, assessment of bladder and bowel dysfunction, genetic counseling, cascade testing of at-risk relatives, and individualized endocrine evaluation when clinically indicated [2].

Several limitations should be acknowledged. C26:0-lysophosphatidylcholine was not documented as having been measured, and biochemical characterization was therefore limited to conventional plasma VLCFA analysis. Because C26:0-lysophosphatidylcholine has superior diagnostic sensitivity, particularly in heterozygous women, the absence of this assay limits the completeness of the biochemical confirmation. However, the elevated C26:0 concentration and increased C24:0/C22:0 and C26:0/C22:0 ratios were concordant with both the compatible clinical phenotype and the pathogenic ABCD1 variant, providing convergent support for the diagnosis. The follow-up period was limited to approximately 25 months from symptom onset and approximately six months from molecular confirmation, precluding a robust assessment of long-term neurological progression. Family segregation analysis was not performed; although genetic counseling and cascade testing were discussed, segregation data were unavailable at the time of manuscript preparation. Standardized longitudinal functional measures, including the Expanded Disability Status Scale, Spastic Paraplegia Rating Scale, modified Rankin Scale, and timed gait testing, were not systematically collected. In addition, a dedicated hereditary spastic paraplegia panel and cerebrospinal fluid analysis were not performed, limiting the complete exclusion of selected hereditary and inflammatory myelopathies. Brain MRI demonstrated nonspecific white matter hyperintensities rather than a characteristic cerebral X-ALD pattern; therefore, these abnormalities cannot be confidently attributed to cerebral involvement without formal neuroradiological review and standardized imaging assessment. Finally, as a single case report, this study cannot establish a genotype-phenotype correlation or estimate the frequency of symptomatic ABCD1-related disease among women in Colombia, particularly because the clinical course of X-ALD cannot be reliably predicted from the specific ABCD1 variant alone [11].

## Conclusions

ABCD1-related disease should be considered among the differential diagnoses of adult women with chronic noncompressive myelopathy or upper motor neuron-predominant ALS/primary-lateral-sclerosis-like presentations, particularly when serial electrodiagnostic studies do not fulfill ALS criteria. Biochemical and molecular confirmation is essential to avoid misclassification as ALS and to redirect care toward symptomatic management, rehabilitation, genetic counseling, cascade testing, and longitudinal surveillance.

## Appendices

**Table 2:** Supplementary Material 1. Detailed electrodiagnostic and neuromuscular ultrasound findings from the September 11, 2025 evaluation Supplementary Material 1: Detailed electrodiagnostic and neuromuscular ultrasound findings from the September 11, 2025 evaluation. Motor and sensory nerve conduction studies showed distal latencies, amplitudes, and conduction velocities within the laboratory reference ranges. F-wave latencies were normal in all tested nerves. Needle electromyography sampled bulbar, cervical, thoracic, and lumbosacral regions and demonstrated normal insertional activity, absence of fibrillation potentials and positive sharp waves, normal motor unit action potential morphology, and normal recruitment and interference patterns. Neuromuscular ultrasound showed peripheral nerve cross-sectional areas within the corresponding reference limits, a total UPS-A score of 0, and no sonographic fasciculations or abnormal muscle echogenicity. Overall, the study provided no neurophysiological or ultrasonographic evidence of lower motor neuron or peripheral nerve involvement. CMAP, compound muscle action potential; CSA, cross-sectional area; MUAP, motor unit action potential; P–T, peak-to-trough; UPS-A, Ultrasound Pattern Sum Score A; CN, cranial nerve.

Study component	Nerve / side	Recording, stimulation, or muscle site	Findings	Reference values
Sensory nerve conduction studies				
Sensory NCS	Left median sensory	2nd digit	Peak latency: 2.2 ms P–T amplitude: 57.3 µV Distance: 14.0 cm Conduction velocity: 64 m/s	Peak latency: <3.6 ms P–T amplitude: >10 µV Conduction velocity: >39 m/s
Sensory NCS	Right median sensory	2nd digit	Peak latency: 3.0 ms P–T amplitude: 72.0 µV Distance: 14.0 cm Conduction velocity: 47 m/s	Peak latency: <3.6 ms P–T amplitude: >10 µV Conduction velocity: >39 m/s
Sensory NCS	Left superficial peroneal sensory	Anterolateral malleolus	Peak latency: 3.1 ms P–T amplitude: 17.6 µV Distance: 14.0 cm Conduction velocity: 45 m/s	Peak latency: <4.0 ms P–T amplitude: >5.0 µV Conduction velocity: >32 m/s
Sensory NCS	Right superficial peroneal sensory	Anterolateral malleolus	Peak latency: 3.0 ms P–T amplitude: 25.5 µV Distance: 14.0 cm Conduction velocity: 47 m/s	Peak latency: <4.0 ms P–T amplitude: >5.0 µV Conduction velocity: >32 m/s
Sensory NCS	Right ulnar sensory	5th digit	Peak latency: 3.1 ms P–T amplitude: 52.7 µV Distance: 14.0 cm Conduction velocity: 45 m/s	Peak latency: <3.7 ms P–T amplitude: >15.0 µV Conduction velocity: >38 m/s
Motor nerve conduction studies				
Motor NCS	Right median	Wrist	Onset latency: 3.2 ms CMAP amplitude: 12.0 mV Segment: - Distance: - Conduction velocity: -	Onset latency: <4.2 ms CMAP amplitude: >5 mV Conduction velocity: -
Motor NCS	Right median	Elbow	Onset latency: 6.8 ms CMAP amplitude: 12.4 mV Segment: Elbow-wrist Distance: 23.0 cm Conduction velocity: 64 m/s	Onset latency: - CMAP amplitude: - Conduction velocity: >50 m/s
Motor NCS	Right peroneal	Ankle	Onset latency: 4.8 ms CMAP amplitude: 5.6 mV Segment: - Distance: - Conduction velocity: -	Onset latency: <6.1 ms CMAP amplitude: >2.5 mV Conduction velocity: -
Motor NCS	Right peroneal	Below fibular head	Onset latency: 11.0 ms CMAP amplitude: 4.9 mV Segment: Below fibular head-ankle Distance: 30.0 cm Conduction velocity: 48 m/s	Onset latency: - CMAP amplitude: - Conduction velocity: >38 m/s
Motor NCS	Left tibial	Ankle	Onset latency: 5.2 ms CMAP amplitude: 18.4 mV Segment: - Distance: - Conduction velocity: -	Onset latency: <6.1 ms CMAP amplitude: >3.0 mV Conduction velocity: -
Motor NCS	Left tibial	Knee	Onset latency: 11.8 ms CMAP amplitude: 13.2 mV Segment: Knee-ankle Distance: 42.0 cm Conduction velocity: 64 m/s	Onset latency: - CMAP amplitude: - Conduction velocity: >35 m/s
Motor NCS	Left ulnar	Wrist	Onset latency: 2.9 ms CMAP amplitude: 11.3 mV Segment: - Distance: - Conduction velocity: -	Onset latency: <4.2 ms CMAP amplitude: >5 mV Conduction velocity: -
Motor NCS	Left ulnar	Below elbow	Onset latency: 7.3 ms CMAP amplitude: 10.2 mV Segment: Below elbow-wrist Distance: 26.0 cm Conduction velocity: 59 m/s	Onset latency: - CMAP amplitude: - Conduction velocity: >53 m/s
F-wave studies				
F-wave	Right median	F-wave	F-wave latency: 23.76 ms	Upper limit: <33 ms
F-wave	Right peroneal	F-wave	F-wave latency: 45.72 ms	Upper limit: <60 ms
F-wave	Left tibial	F-wave	F-wave latency: 49.11 ms	Upper limit: <61 ms
F-wave	Left ulnar	F-wave	F-wave latency: 24.24 ms	Upper limit: <36 ms
Needle electromyography (EMG)				
Side Muscle Nerve Root Ins.Act Fibs Psw Amp Dur Poly Recrt Int.Pat Comment Bilateral FDI Ulnar C8-T1 Nml None None Nml Nml 0 Nml Nml Left Ext indicis Radial/post. interosseous C7-C8 Nml None None Nml Nml 0 Nml Nml Bilateral Biceps Musculocut. C5-C6 Nml None None Nml Nml 0 Nml Nml Right Deltoid Axillary C5-C6 Nml None None Nml Nml 0 Nml Nml Bilateral AntTibialis Deep peroneal L4-L5 Nml None None Nml Nml 0 Nml Nml Bilateral Gastroc Tibial S1-S2 Nml None None Nml Nml 0 Nml Nml Bilateral VastusMed Femoral L2-L4 Nml None None Nml Nml 0 Nml Nml Bilateral Hyoglossus Hypoglossal CN XII Nml None None Nml Nml 0 Nml Nml Right SCM Spinal accessory CN XI, C2-C3 Nml None None Nml Nml 0 Nml Nml Bilateral Thoracic PS Rami T5-T9 Nml None None Nml Nml 0 Nml Nml EMG abbreviations: FDI, first dorsal interosseous; SCM, sternocleidomastoid; PS, paraspinals; Nml, normal; Ins.Act, insertional activity; Fibs, fibrillation potentials; Psw, positive sharp waves; Amp, MUAP amplitude; Dur, MUAP duration; Poly, polyphasia; Recrt, recruitment; Int.Pat, interference pattern.				
Peripheral nerve ultrasound				
Nerve ultrasound	Median nerve	Forearm	CSA: 7 mm² UPS-A score: 0	Reference upper limit: 10.7 mm²
Nerve ultrasound	Median nerve	Elbow	CSA: 9 mm² UPS-A score: 0	Reference upper limit: 13.2 mm²
Nerve ultrasound	Median nerve	Arm	CSA: 9 mm² UPS-A score: 0	Reference upper limit: 13.1 mm²
Nerve ultrasound	Ulnar nerve	Forearm	CSA: 6 mm² UPS-A score: 0	Reference upper limit: 8.3 mm²
Nerve ultrasound	Ulnar nerve	Arm	CSA: 6 mm² UPS-A score: 0	Reference upper limit: 7.9 mm²
Nerve ultrasound	Fibular nerve	Fibular head	CSA: 14 mm² UPS-A score: 0	Reference upper limit: 17.8 mm²
Nerve ultrasound	Tibial nerve	Knee	CSA: 36 mm² UPS-A score: 0	Reference upper limit: 55.9 mm²
Nerve ultrasound	Tibial nerve	Ankle	CSA: 15 mm² UPS-A score: 0	Reference upper limit: 22.3 mm²
Nerve ultrasound	Total	-	Total UPS-A score: 0	-
